# Invasive Pneumococcal Disease and COVID-19 Coinfection: A Series of Cases Admitted to an Intensive Care Unit

**DOI:** 10.7759/cureus.31876

**Published:** 2022-11-25

**Authors:** Manuel Almeida, Pedro Lavado, Lucie Cunha, Isa Cordeiro, Alexandre Baptista

**Affiliations:** 1 Intensive Care Unit, Centro Hospitalar Universitário do Algarve, Portimão, PRT

**Keywords:** risk factors, sars-cov-2, pneumonia, streptococcus pneumoniae, invasive pneumococcal disease

## Abstract

Pneumococcal infection is still a frequent disease. It can be classified as invasive when pneumococcus is isolated in a generally sterile fluid. Pneumonia is the most common infectious source of adult invasive pneumococcal disease (IPD), and several risk factors for IPD are well known. This case report presents three clinical cases of different manifestations of IPD. The two most severe cases had coinfection by SARS-CoV-2 at hospital admission.

## Introduction

Invasive pneumococcal disease (IPD) is an infection caused by Streptococcus pneumoniae (pneumococcus); it is defined by the isolation of pneumococcus in a generally sterile fluid such as blood, cerebrospinal fluid, pleural fluid, or synovial fluid. Bacteremia is most often associated with pneumonia, occurring regardless of the immune status of the patient [1]. The majority of cases were reported in males, with a male-to-female ratio of 1.2:1 [2]. According to the 2018 annual epidemiological report of the European Centre for Disease Prevention and Control (ECDC), Portugal had 397 confirmed cases of IPD out of a total of 24,663 confirmed cases in 29 countries of the European Economic Area [2].

Some risk factors for infection are described in the literature, such as functional or anatomical asplenia, chronic pulmonary disease, chronic cardiovascular disease, chronic kidney disease, chronic liver disease, diabetes, immunosuppression, individuals with cochlear implants, and with cerebrospinal fluid leaks [3]. The initial diagnostic approach to community-acquired pneumonia includes urinary antigen tests for pneumococcus, with a described sensitivity of approximately 74% and a specificity of 97.2% [4]. A positive urine pneumococcal antigen test is associated with more severe illness, including the need for intensive care admission, therapeutic failure, and increased mortality in 30 days [5]. Definitive diagnosis for IPD requires identification of pneumococcus in blood culture or other sterile fluids, as described above. In this report, we present three clinical cases managed in a peripheral Portuguese intensive care unit (ICU).

## Case presentation

Case 1

**Figure 1:**
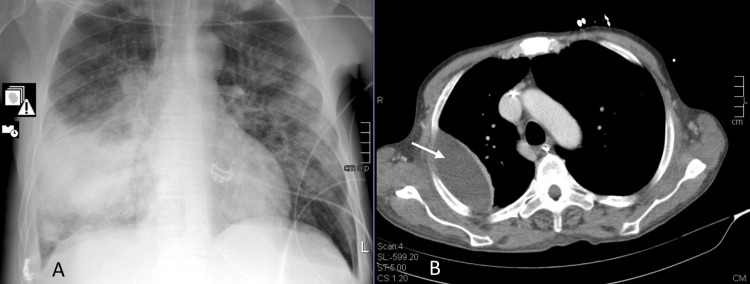
X-ray and CT scan of the patient in case 1 A: X-ray at admission – right middle and lower consolidation. B: CT scan made on day 15 of hospital stay showing one of the lung abscesses later drained.

We present the case of a 54-year-old homeless male with a history of inhaled cocaine, alcohol, and tobacco (30 pack-years) abuse. He never fulfilled the criteria for receiving the anti-pneumococcal vaccine and was unvaccinated against SARS-CoV-2. The patient was admitted to the ICU with a septic shock of pulmonary origin. Lobar pneumonia was diagnosed (Figure 1A) with the following severity indices for community-acquired pneumonia: score for pneumonia severity risk of death at 30 days (CURB-65) of four and pneumonia severity index (PSI) of 179 (risk class V). SARS-CoV-2 coinfection was identified. He had a urinary antigen test positive for pneumococcus and blood cultures positive for a multisensitive Streptococcus pneumoniae. During the ICU stay, cardiovascular, respiratory, and hematological dysfunctions were present. Clinical evolution was unfavorable, with the development of necrotizing pneumonia with multiple pulmonary abscesses, as displayed in Figure 1B. Computed tomography-guided catheter drainage was performed. After consulting thoracic surgery, no surgical indication was identified, and the patient was kept on prolonged antibiotic therapy. Other identified complications included Strenotrophomonas maltophilia bacteremia following ventilator-associated pneumonia (VAP) and intensive care unit-acquired weakness. The patient stayed in the ICU for 44 days and completed 35 days of ceftriaxone. The suspension of antibiotic therapy was based on imagiological improvement and in agreement with the antimicrobial stewardship team. When VAP was suspected, piperacillin and tazobactam were started and de-escalated to trimethoprim-sulfamethoxazole after sensitivity tests. The patient was then transferred to the internal medicine ward for maintenance of care and functional rehabilitation. He was discharged to a rehabilitation facility on the 101st day of hospitalization due to his functional limitation secondary to ICU-acquired weakness. Two months after discharge, the patient was readmitted to the hospital with a hemorrhagic shock from oesophageal variceal bleeding, the first presentation of an underlying chronic liver disease. The hemorrhage stopped after a gastroenterological intervention. No recurrence of respiratory symptoms was observed.

Case 2

**Figure 2:**
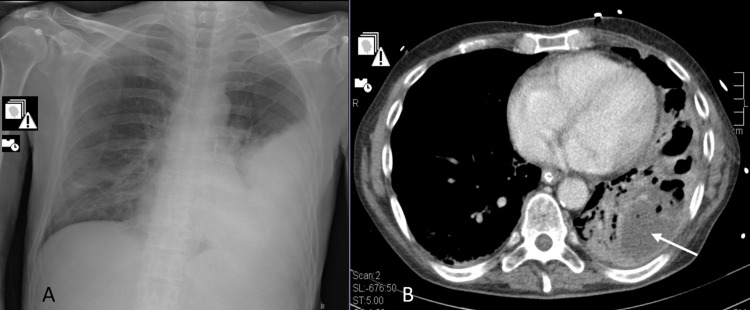
X-ray and CT scan of the patient in case 2 A: X-ray at admission - left lower consolidation. B: CT scan made on day 10 of hospital stay showing a lung abscess and cavitation.

We present another case of a 56-year-old male who had an unstratified liver disease and a history of alcohol abuse. He never fulfilled the criteria for receiving the anti-pneumococcal vaccine and was unvaccinated against SARS-CoV-2. The patient was admitted to the ICU with a septic shock of pulmonary origin. Severe lobar pneumonia (Figure 2A) was diagnosed (CURB-65 of four and PSI of 171, risk class V), as well as SARS-CoV-2 coinfection. He had a urinary antigen test positive for pneumococcus and blood cultures positive for a multisensitive Streptococcus pneumoniae. During the ICU stay, cardiovascular, respiratory, and hematological dysfunctions were present. Clinical evolution was unfavorable with the development of necrotizing pneumonia with multiple pulmonary abscesses (Figure 2B). Computed tomography-guided catheter drainage was performed. There was a discussion about this patient with the thoracic surgery department, with no surgical indication and recommendation for prolonged antibiotic therapy. The patient remained in the ICU for 44 days, receiving a total of 33 days of antibiotic therapy with ceftriaxone. The suspension of antibiotic therapy was also based on imagiological improvement and in agreement with the antimicrobial stewardship team. He was subsequently transferred to the internal medicine department for maintenance of care and continuing functional rehabilitation and was discharged to a rehabilitation facility on the 52nd day of hospitalization due to his functional limitation. No recurrence of respiratory symptoms or any other complication were observed in the follow-up consultations.

Case 3

**Figure 3:**
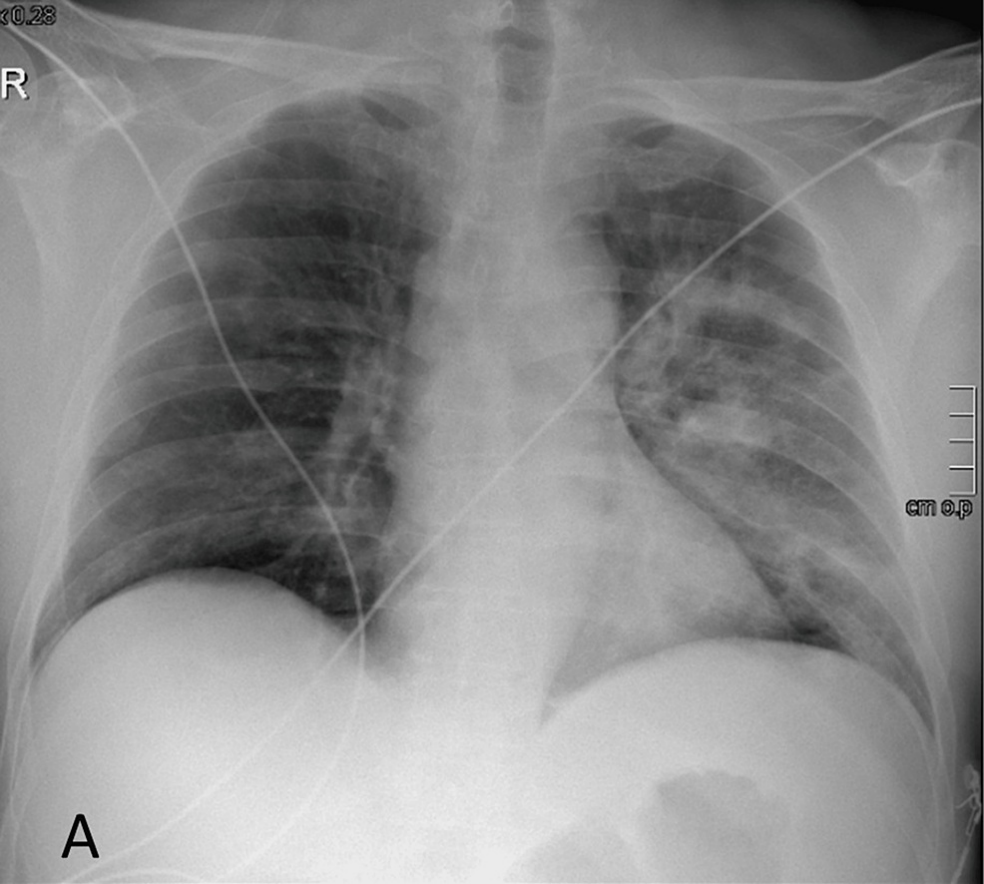
X-ray of the patient in case 3 X-ray at presentation showing left alveolar infiltrates.

The last patient of this series was a 57-year-old male. His medical history included untreated arterial hypertension and osteoarthritis. He was a former smoker (60 pack-years) and had active alcohol consumption. He never fulfilled the criteria for receiving the anti-pneumococcal vaccine but was fully vaccinated against SARS-CoV-2. He was admitted to the ICU with acute hypoxemic respiratory failure due to community-acquired pneumonia (Figure 3; CURB-65 of two and PSI of 127). He had a urinary antigen test positive for pneumococcus and blood cultures positive for a multisensitive Streptococcus pneumoniae. Unlike the other two cases, this patient had a favorable clinical progression, with a short oxygen weaning period. He remained in the ICU for four days and was later transferred to a medical ward. He was discharged after 12 days of ceftriaxone. He continued to follow up with his family's doctor, and there was no record of complications.

Table 1 summarizes the clinical findings from the three patients admitted to our ICU. Informed consent was obtained from all patients.

**Table 1:** Case presentations of the patients CURB-65 - score for pneumonia severity risk of death at 30 days increases as the score increases, it varies from 0 to 5; PSI - pneumonia severity index to classify the severity of a patient's pneumonia to determine the amount of resources to be allocated for the care, it stratifies the risk from I to V; RC - risk class; VAP - ventilator-associated pneumonia; ICU - intensive care unit * Suspension of antibiotic therapy was based on imagiological improvement and in agreement with the antimicrobial stewardship team. # Decision depending on the presence or absence of activity limitation secondary to ICU-acquired weakness.

	Case 1: 54 years old ♂	Case 2: 56 years old ♂	Case 3: 57 years old ♂
Medical history	Inhaled cocaine, alcohol, and tobacco (30 pack-year) abuse	Unstratified liver disease and alcohol abuse	Untreated arterial hypertension and osteoarthritis; former smoker (60 pack-year) and had an active alcohol consumption
Anti-pneumococcal vaccine/SARS-CoV-2 vaccine	-/-	-/-	-/+
CURB-65/PSI (risk class)	4/179 (RC V)	4/171 (RC V)	2/127 (RC IV)
SARS-CoV-2 coinfection	+	+	-
Urinary antigen test for pneumococcus	+	+	+
Blood culture	Streptococcus pneumoniae multisensitive	Streptococcus pneumoniae multisensitive	Streptococcus pneumoniae multisensitive
Organ dysfunctions	Cardiovascular, respiratory, hematological	Cardiovascular, respiratory, hematological	Respiratory
Complications during the hospital stay	Necrotizing pneumonia with multiple pulmonary abscesses needing drainage; Strenotrophomonas maltophilia bacteremia following VAP; intensive care unit-acquired weakness	Necrotizing pneumonia with multiple pulmonary abscesses needing drainage; intensive care unit-acquired weakness	None
Antibiotics^*^	35 days of ceftriaxone. When VAP was suspected, piperacillin and tazobactam and then de-escalated to trimethoprim-sulfamethoxazole completing seven days	33 days of ceftriaxone	12 days of ceftriaxone
Length of stay	44 days in ICU, 101 days of hospitalization	44 days in ICU, 52 days of hospitalization	4 days in ICU, 12 days of hospitalization
Discharged to^#^	Rehabilitation facility	Rehabilitation facility	Home
Complications after three months of hospital discharge	Hemorrhagic shock from oesophageal variceal bleeding, as the first presentation of underlying chronic liver disease	None	None

## Discussion

This paper presents three clinical cases of different manifestations of IPD. The two most severe cases presented coinfection by SARS-CoV-2 at hospital admission, with none of these patients vaccinated for SARS-CoV-2. There are some reported cases of pneumococcal bacterial coinfection with COVID-19 [6]. However, literature on the association between IPD and COVID-19 is scarce. When IPD and COVID-19 are present, mortality is much higher, particularly in the elderly and frail people [7]. In our case series, the only patient without coinfection had a much more benign course of illness, lacking any of the complications present in the other two cases. Keeping in mind that COVID-19 may cause vasculitis, immune deregulation, and alveolar damage, we may hypothesize that this could be one of the reasons that can lead to a more severe pneumococcal disease.

The risk factors associated with mortality in IPD are advanced age, being a nursing home resident, concomitant nosocomial infection, septic shock at admission, solid neoplasms, immunosuppressive states, and chronic illness (e.g., diabetes mellitus, chronic obstructive pulmonary disease, human immunodeficiency virus infection) [8]. Considering the description of these cases, we can suggest that COVID-19 may stand as a new risk factor for severe infection despite the age of the patient.

When a patient fulfills clinical criteria (although it may vary from country to country), vaccination should be offered. We hypothesize that two of our previously described patients, who had a worse clinical progression, could have had clinical criteria for being vaccinated, given their possible undiagnosed/non-stratified chronic liver disease. Despite the adoption of anti-pneumococcal vaccination in individuals with an increased risk of IPD, the mortality rate remains high at around 20.8% [8]. On the other hand, pneumococcal pneumonias with bacteremia are associated with higher in-hospital mortality, as well as longer hospital stays, compared to patients without bacteremia [9]. A review study showed that patients with IPD of pulmonary origin had higher values of C-reactive protein and procalcitonin, as well as higher levels of pro- and anti-inflammatory cytokines, in particular Interleukin 6 (IL-6) [10].

The most common complications associated with IPD of pulmonary origin are empyema and lung abscess. In the presence of such complications, a source control strategy, as well as prolonged antibiotic therapy, are the mainstays of treatment. Such an approach is associated with lower morbidity and mortality rates. As for the antibiotic treatment, empirical combination therapy of a β-lactam and a macrolide is recommended and should be adjusted whenever microbiological and antibiotic sensitivity results are available [11]. The reason for disease progression and whether pleural invasion occurs either by local extension or by hematogenic origin is not completely understood [12]. Mortality is higher if other infections are present (i.e., meningitis, endocarditis, pericarditis, septic arthritis, and peritonitis) [13].

We found no studies in the literature showing the ideal duration of antibiotic therapy in the treatment of IPD. In uncomplicated bacteremia, referring to immunocompetent patients without an uncontrolled source of infection, a 10 to 14 days regimen may be adequate. Some factors should be considered before opting for a longer-duration regimen of antibiotic therapy, such as the site of infection, the immunological status of the patient, the presence of pulmonary abscesses, and the patient's response to the treatment. In the presence of pulmonary abscesses, as seen in two of our patients, antibiotic therapy should be prolonged, although there is no specific number of days; literature shows treatment regimens between 21 and 48 days [14,15]. The consensus is that antibiotic therapy should be maintained until source control is achieved and lung images are improved. The former point was the main reason for our antibiotic strategy in the two most severe cases. Surgical intervention (empyema excision, decortication, lobectomy, or segmentectomy) is reserved for cases in which there is no clinical improvement, the impossibility of non-surgical drainage, or the presence of multiloculated abscesses [16].

The anti-pneumococcal vaccine has proven to be the best strategy for the prevention of pneumococcal infection and, therefore, IPD [17,18]. In a review comparing COVID-19 vaccines, one of the conclusions was that vaccines and complete immunization offered protection against more severe cases of COVID-19 and reduced the number of hospitalizations [19]. This way, avoiding a more severe infection of COVID-19 could act as a protective factor for IPD.

## Conclusions

Pneumococcal infection is still a frequent disease. Complications and mortality vary according to whether the disease is invasive or non-invasive. Pneumonia is the most common infectious source of IPD in adults. Several risk factors for IPD are well known, and COVID-19 should be taken into account as a risk factor for severe IPD, as shown by the evolution of our patients. Clinicians should be aware that this coinfection can have worse outcomes, and these patients should be discussed with the ICU team along with close monitoring. Clinical cases are scattered, and further investigation is needed in order to understand the pathophysiology and relationship between IPD and COVID-19, as well as why IPD develops.
